# Patient safety in Dutch primary care: a study protocol

**DOI:** 10.1186/1748-5908-5-50

**Published:** 2010-06-28

**Authors:** Mirjam Harmsen, Sander Gaal, Simone van Dulmen, Eimert de Feijter, Paul Giesen, Annelies Jacobs, Lucie Martijn, Theodorus Mettes, Wim Verstappen, Ria Nijhuis-van der Sanden, Michel Wensing

**Affiliations:** 1 Scientific Institute for Quality of Healthcare, Radboud University Nijmegen Medical Centre, Nijmegen, The Netherlands; 2 Department of Oral Sciences, Preventive and Operative Dentistry, Radboud University Nijmegen Medical Centre, Nijmegen, The Netherlands

## Abstract

**Background:**

Insight into the frequency and seriousness of potentially unsafe situations may be the first step towards improving patient safety. Most patient safety attention has been paid to patient safety in hospitals. However, in many countries, patients receive most of their healthcare in primary care settings. There is little concrete information about patient safety in primary care in the Netherlands. The overall aim of this study was to provide insight into the current patient safety issues in Dutch general practices, out-of-hours primary care centres, general dental practices, midwifery practices, and allied healthcare practices. The objectives of this study are: to determine the frequency, type, impact, and causes of incidents found in the records of primary care patients; to determine the type, impact, and causes of incidents reported by Dutch healthcare professionals; and to provide insight into patient safety management in primary care practices.

**Design and methods:**

The study consists of three parts: a retrospective patient record study of 1,000 records per practice type was conducted to determine the frequency, type, impact, and causes of incidents found in the records of primary care patients (objective one); a prospective component concerns an incident-reporting study in each of the participating practices, during two successive weeks, to determine the type, impact, and causes of incidents reported by Dutch healthcare professionals (objective two); to provide insight into patient safety management in Dutch primary care practices (objective three), we surveyed organizational and cultural items relating to patient safety. We analysed the incidents found in the retrospective patient record study and the prospective incident-reporting study by type of incident, causes (Eindhoven Classification Model), actual harm (severity-of-outcome domain of the International Taxonomy of Medical Errors in Primary Care), and probability of severe harm or death.

**Discussion:**

To estimate the frequency of incidents was difficult. Much depended on the accuracy of the patient records and the professionals' consensus about which types of adverse events have to be recognized as incidents.

## Background

*Primum non nocere *('first do no harm') has been a maxim of healthcare workers for many centuries. In the past decade, patient safety has been placed high on the societal agenda. This can be seen from high-profile cases of compromised patient safety around the world, policy reports such as *To err is human *in the United States [1], a growing overall aversion of risk in society, and the fact that healthcare professionals have started to realize that there is a lot to gain in the quality of care by focussing explicitly and systematically on patient safety.

There are many definitions of patient safety and unsafety. The World Health Organisation defines patient unsafety as *a process or act of omission or commission that resulted in hazardous healthcare conditions and/or unintended harm to the patient *[2]. Wagner and Van der Wal [3] define a patient safety incident as *an unintended event during the care process that resulted, could have resulted or still might result in harm to the patient*. A more specific unit used in this type of research is the adverse event. Zegers *et al*. [4] define an adverse event as *an unintended injury that results in temporary or permanent disability, death or prolonged hospital stay, and is caused by healthcare management rather than by the patient's underlying disease process*.

Research into patient safety can be positioned in the broader field of implementation science. When an adverse event has occurred (*e.g.*, the patient died during treatment), a significant event analysis has to be made to determine the preventability of this adverse event. When a clinical decision is not consistent with the recommended procedures (*e.g.*, a clinical guideline or professional standard was not followed), an analysis has to be made to determine the actual risk for adverse outcomes. In both cases, the assessment of patients' safety can only be made on the basis of scientific knowledge, integrated with clinical expertise, about the relation between clinical decisions or practices (*e.g.*, prescribing medication), and adverse outcomes (*e.g.*, worsening of symptoms or prolonged illness). Therefore, insight into the frequency and seriousness of potentially unsafe situations may be the first step towards improving patient safety.

Most attention to patient safety has been directed at hospitals, because hospital care clearly implies high-risk procedures (*e.g.*, surgery and blood transfusion) and a riskful environment (*e.g.*, hospital-acquired infections and pressure ulcers). According to national and international studies, 3% to 17% of the patients in acute care hospitals have one or more adverse events. Patients die due to 5% to 13% of the adverse events [4-6]. Approximately 50% of the adverse events are considered potentially preventable [4]. A Dutch costing study has shown that estimates indicate that the total of preventable direct medical costs of adverse events in hospitals form a substantial part (1%) of the expenses of the national healthcare budget. The expenses are mainly due to an excessively long stay (including readmissions) [5].

Hospital care, although important, represents only a fraction of a patient's use of the healthcare services [7]. In many countries, including the Netherlands, most patients receive most of their healthcare in primary care settings. Although primary care may imply lower risks for the patient, the large volume of contacts and procedures in this healthcare system implies that incidents can be expected to occur in primary care. For instance, one of the characteristics of primary healthcare is multidisciplinary co-working (*e.g.*, general practitioner (GP) and physiotherapist, general dental practitioner (GDP) and dental hygienist), which implies extended communication and consequences for transferring information.

There are also studies of patient safety that show that incidents in hospital care have their origin in primary care. For example, the Dutch HARM (Hospital Admissions Related to Medication) study showed that the cause of unintended hospital admissions were medication errors in extramural care (*i.e.*, primary care and out-patient clinics) [8]. A French national study of adverse events in 2004 revealed that 3.5% of admissions to general medicine departments and 4.5% of admissions to surgical departments were due to events occurring outside the hospital [9]. An English study of 18,820 patients admitted to hospital showed that 6.5% of these admissions were related to adverse drug reactions. Although most patients recovered, 28 (2.3%) died as a direct result of the index adverse drug reaction (as detailed in either the case notes or on the death certificate) [10]. A German incident-reporting system for general practices ('Jeder Fehler Zählt') received 188 classifiable reports in the 17 months following its launch in September 2004; 41.5% of these reports were associated with harm to the patient [11]. Errors and preventable adverse events were identified in 24% of 351 outpatient visits in the USA. Harm was believed to have occurred as a result of 24% of the errors, and there was potential harm in another 70% [12]. Note that the patient populations and methods differed, which may have influenced the numbers. For instance, in a French hospital study [9], patients were actually observed, while the German data [11] were based on a reporting system.

There are, however, scant data about patient safety in primary care in the Netherlands. In a small-scale study in two Dutch general practices, GPs recorded all the adverse events they encountered in their regular office hours during an observation period of five months. During this period, 4,095 patients visited the practice, and a total of 31 adverse events were noted (0.7%). About one-half of the events did not have health consequences, but one-third led to worsening of symptoms, and a few resulted in unplanned hospital admissions [13]. A cross-sectional, multicentre, observational study employed five coached patients who telephoned the triage nurses of four Dutch GP cooperatives. The study shows that the triage nurses estimated the level of urgency of 69% of the 352 contacts correctly. They underestimated the level of urgency of 19% of the contacts [14].

In allied healthcare, some incidents resulting in harm to or even death of children are mentioned in the Netherlands and internationally [15-17]. There are also some studies of incidents with spinal procedures of adults. Dissection of the vertebral arteries was the most common problem; other complications included dural tear, oedema, nerve injury, disc herniation, haematoma, and bone fracture. The symptoms were frequently life-threatening, though in most cases the patient fully recovered. In most cases, a spinal procedure was deemed to be the probable cause of the adverse effect [18-20].

There are hardly any other data about the incidence of incidents in primary healthcare settings in the Netherlands [21].

## Aims and objectives

Current data regarding patient safety in primary care in the Netherlands are needed to identify performance gaps (both under- and over-treatment) and underlying factors, to tailor interventions to deal with the relevant obstacles to and enablers for change, and to set specific targets for improvement. The Dutch Ministry of Health, Welfare, and Sport has developed a policy to improve safety in healthcare, including primary care, and has called for a study to describe the situation at the start of this policy programme.

This study protocol concerns a study of patient safety in primary care practices (general practices), out-of-hours primary care centres, general dental practices, midwifery practices, and allied healthcare practices (with physiotherapists, occupational therapists, and/or Cesar-Mensendieck therapists). The overall aim was to provide insight into current patient safety issues. Such insight would help inform national health policy makers and decision makers in the domain. The objectives of this study were: to determine the frequency, type, impact, and causes of incidents found in the records of Dutch primary care patients; to determine the type, impact, and causes of incidents reported by healthcare professionals; and to provide insight into safety management in primary care practices by means of a written survey.

## Definitions

Because we did not want to focus only on events that actually caused harm, we used a broader definition of 'incident': an unintended event during the care process that resulted, could have resulted, or still might result in harm to the patient [3].

However, this is a very broad definition indeed, and it is difficult to use in specific primary healthcare settings. Gaal *et al.*'s study [22], based on a web-based survey of 68 general practices, shows that the clinical cases were not uniformly judged as particularly safe or unsafe.

On the basis of our reading of the literature and discussions in the project team, we presented the following description of a patient safety event. We considered both acts of omission and of commission, although not everyone on the project team would consider acts of omission always necessarily a threat to patient safety. We included incidents related to unnecessary harm or risk to the individual patient. We thought of the harm as somatic (*e.g.*, death, pain, infection, and injuries), but included serious psychiatric or mental diseases (*e.g.*, anxiety disorder and stress responses) as well. In cases of risk of harm to the patient (rather than actual harm, such as prolonged recovery), we agreed that the risk had to be scientifically proven or broadly accepted as valid (*e.g.*, by recommendations in guidelines). Patients can contribute to incidents, but we exclude incidents that are completely caused by a patient (*e.g.*, not adhering to therapy). We do not use other terminology, such as adverse events, or near incidents.

We tested our definition in a pilot study, and proved it to be functional. Fifty patient records from each study were judged by at least two reviewers. The proportion of agreement about whether an event should be defined as a patient safety incident was good to very good, varying from 75% (midwifery care) to 100% (out-of-hours primary care).

## Hypothesis

While the study is mainly descriptive and explorative, we formulated the following hypothesis: patient safety in primary care is relatively good, meaning that fewer incidents per 100,000 contacts occur in primary care than in hospital care, and fewer of these incidents have major adverse outcomes.

## Design and methods

An observational study of patient safety in primary care has shown that a mix of methods is needed to identify incidents in general practice [23]. Therefore, the current study has a retrospective component and a prospective one. The retrospective component concerns a patient record study and a written survey of health professionals. The prospective design concerns an incident-reporting study. Table 1 illustrates the framework for the study.

**Table 1:** Overview of methods and outcome measures

**Objective 1: To determine the frequency, type, impact, and causes of incidents affecting primary care patients**
Method: retrospective patient record study
Outcome measures: practice type, patient sex, patient age (category), social status of patient, recording of possible communication problems, patient's risk, number of contacts in study year, urgency of the request for help, having seen health professional(s) outside the practice for the same health problem, accuracy of record keeping, question of whether the event was an incident, description of the incident, action(s) taken afterwards.
Analysis of incidents: type of incident, cause (by Eindhoven Classification Model class [27]), actual harm (by the severity-of-outcome domain of the International Taxonomy of Medical Errors in Primary Care [32]), probability of severe harm or death (as judged by the reviewers).

**Objective 2: To determine the type, impact, and causes of incidents reported by healthcare professionals**
Method: prospective incident-reporting study.
Outcome measures: information about the reporting person (*e.g.*, function), patient's year of birth. patient's sex, description of the incident, action(s) taken afterwards, possible consequences of the incident, and suggestions how to prevent similar incidents in the future.
Analysis of incidents: type of incident, cause (by Eindhoven Classification Model class [27]), actual harm (as defined by the severity-of-outcome domain of the International Taxonomy of Medical Errors in Primary Care [32]), probability of severe harm or death (as judged by the reviewers).

**Objective 3: To get insight into the patient safety management of primary care practices**
Method: written survey
Outcome measures:
Practice characteristics (practice type, number of health professionals in the practice, proportion of patients < 75 years old, proportion of patients with low social status, mean number of hours of patient contacts and management tasks per week, and whether the practice has an educational function);
Topics related to quality and safety management (*e.g.*, existence of joint policy, annual report, quality aspects of the annual report, policy plan, quality system, standard procedure for complaints, registration of incidents and near incidents, and method of processing digital data);
Safety culture of the practice (*e.g.*, is it easy to discuss incidents within the practice, learn from each other's mistakes, express concerns about patient care, ask questions for clarity, correct follow-up of incidents, and report concerns about patient safety?).

### Setting

The setting is one of practices, health professionals, and patient records in primary healthcare in the Netherlands.

#### Practices

Separate studies were carried out in general practices, out-of-hours primary care centres, general dental practices, midwifery practices, and allied healthcare practices (with physiotherapists, occupational therapists, and/or Cesar-Mensendieck therapists). Stratified random sampling of 20 practices was performed for each study, except for the out-of-hours primary care study. Twenty general practices related to four centres (five practices for each centre) were selected for the study of out-of-hours primary care centres. We chose a sample size of 20 practices for each study because it was feasible in the context and budget of the project, and experience has shown that this sample size is large enough to give reliable results.

For a stratified random sample, we used two factors for stratification: practice size and urbanization. We defined a small practice as one with no more than the equivalent of two full-time jobs for primary care health professionals (GPs, *et al*.), and we defined large practices as having more than the equivalent of two full-time jobs (regarding the type of contract and reimbursement) for primary care health professionals. Trainees and nurse practitioners are not included in this definition. The practices may be part of larger organizational networks, such as multidisciplinary health centres or primary care trusts (for instance, for sharing patient lists, financial risk, legal accountability, support staff, *et al*.). This wider organizational context was not considered in the sampling in this project. In this study, 'urban' refers to more than 100,000 inhabitants in the area, while 'rural' or 'town' refers to less than 100,000 inhabitants (considering the geographical location of the practice, although the patients may come from other areas). For reasons of logistics, it is acceptable to sample in one geographical area or a few of them in the country. The degree to which these regions represent the country as a whole is described qualitatively in terms of health system and population health.

There are some exceptions to these sampling rules. In allied healthcare, we stratified the distribution of physical, occupational, and exercise therapy practices. There was no stratification of practice size because occupational and exercise therapy practices are always small.

The practices were compensated for the expenses of their activities at a standardized rate within the project. Depending on the study, accreditation and/or feedback about results was possible.

#### Health professionals

The study considered all staff physically working in each primary care practice, including professionals themselves: GPs, allied healthcare professionals, GDPs, midwives, nurses, practice assistants (with or without clinical tasks), dental hygienists, preventive dental assistants, administrative people, and managers.

#### Patients

There were no restrictions of the type of patients included, except that they had to be registered or be regular practice attendees. They could attend the practice in person, phone the practice, or be visited at home by a health professional. In the patient record study, contacts had to have taken place one to four months before the selection of patient records. Contacts for collecting incidents in the incident-reporting study had to have taken place during two successive weeks.

An exception to this is the study in midwifery practices. The selection was made amongst women who gave birth in 2008. The study also included women who miscarried, had a premature delivery, or only received care in the postnatal period.

### Reviewer recruitment and training

The patient records were reviewed by teams of researchers and, if necessary, health professionals. The reviewers also examined the type and cause of the incidents found in the patient record study and the incident-reporting study. The selection criteria for the reviewers were: at least five years of postgraduate clinical experience (at least one day a week); a retirement of no longer than five years; and experience or affinity with analysis of incidents.

Health professionals were recruited via personal contacts of the project leaders of each substudy.

The reviewers took an e-learning patient-safety course [24], starting with a general introduction to patient safety. One module was compulsory, namely, the PRISMA method module [25,26]. We used this method to classify the causes of the incidents into the Eindhoven Classification Model [27]. The study protocol, definitions, and review forms were explained, and examples of incidents were discussed at meetings. Additionally, the reviewers of each study called as many meetings as necessary to clarify the definition of a patient safety incident within their own fields. A pilot test was also used for this purpose. External reviewers were compensated for their review activities at an hourly rate and for expenses.

### Procedures

We collected data from primary care patient records, incident-reporting forms, and surveys. Table 1 gives an overview of the methods and outcome measures.

#### Patient record study

Fifty patient records were randomly selected from the appointment lists one to four months before the selection date for each sub-study (out-of-hours primary care centres excluded), in each of the 20 practices, for a total of 1,000 patient records. Each record was reviewed by one reviewer from the selection date going back one year to determine whether any incidents occurred in that year. We aimed for great sensitivity, meaning that no incidents were to be missed. Details of each incident that the reviewers found were recorded. The details were discussed with another reviewer within the sub-study in case there was any doubt about whether an event was an incident. If consensus was not achieved, one or more other reviewers provided a final judgement on the basis of information from the other two reviewers.

There were some exceptions to this procedure. Because there were fewer patients and a greater frequency of contacts in allied healthcare practices, and because we wanted to guarantee a random selection, the appointment list of one to twelve months preceding the selection date were used for these practices. The screening period of the record was one year, ending at the selection date. Four GP cooperatives with five practices each were selected for the study of out-of-hours primary care centres. Next, a total of 50 patients who had contact with the GP cooperative at least one week before the selection date were randomly selected from each practice. The patient records in the centre (moment of contact) and in the practice (one week before contact to at least eight weeks after contact with the centre) were reviewed. The end of midwifery care had to be in 2008, and the review period for a pregnancy was nine months. Table 2 shows these procedures.

**Table 2:** Overview of selection and review of patient records

	**General practices**	
T_-1_: 1-4 months before T_0_	**T**_**0**_	
T_-2_: 0-12 months before T_-1_	**T**_**0**_	
	**Out-of-hours primary ** **care centres**	
T_-1_: 1 week before T_0_	**T**_**0**_	
	**T**_**0**_	T_-2_: 1 week before to 8 weeks after T_-1_
	**General dental ** **practices**	
T_-1_: 1-4 months before T_0_	**T**_**0**_	
T_-2_: 0-12 months before T_-1_	**T**_**0**_	
	**Midwifery practices**	
T_-1_: end of midwifery care in 2008	**T**_**0**_	
T_-2_: 0-9 months before T_-1_	**T**_**0**_	
	**Allied healthcare ** **practices**	
T_-1_: 0-12 months before T_0_	**T**_**0**_	
T_-2_: 0-12 months before T_-1_	**T**_**0**_	

T_-2_: review period of patient record, T_-1_: date of patient contact with practice or office, T0: date of actual visit of reviewer to practice or office to select patient records (early 2009)

#### Incident-reporting study

The incident-reporting study was conducted during two successive weeks, and whenever possible, immediately after the patient record study. The health professionals were asked to report all incidents on standardized forms for the patient record study. If no incidents were reported, the practices were asked whether they did not report at all or if they had not encountered any incidents.

Due to practical limits, this procedure was not feasible in the study of out-of-hours primary care centres. For this study, we used prospectively collected information from the incident-reporting systems that the centres were already using.

#### Survey

A questionnaire about organizational and cultural items related to patient safety was sent to a contact person in each practice, but not to the out-of-hours primary care centres. A standard set of questions was designed, and, when necessary, extra questions were added to focus more on the specific topics related to the professional circumstances of the different professions. The contact person was asked to fill in the questionnaire and return it to the research group.

The procedures of the patient record study and the incident-reporting study were tested in a pilot study in six practices. The results were discussed in a plenary meeting of all the researchers in order to standardize the procedures as much as possible. The pilot study shows that the methods and instruments, with some modifications, appeared to combine as the most valid method at hand within the budget and relatively short period available for conducting the study of incidents in primary care.

### Accuracy of figures

The power calculation was based on the patient record study because this method resulted in the most comprehensive overview of patient safety issues. For the moment, we assumed that the number of records with incidents was 30 in every 1,000 records (3%). It is possible that incidents were clustered within individual practices. To what extent this was true was defined as the intracluster correlation (ICC). Assuming an ICC of 0.05 and an alpha of 0.05, the confidence interval becomes 1% to 5%. This is the range in which the 'true' number of incidents will lie in a sample of 1,000 records.

### Measures

Table 1 gives an overview of the methods and outcome measures.

#### Patient record study

For each record, the following items were recorded: practice type, patient gender, patient age (in categories), social status of the patient (determined by checking a list of postal codes of areas with a known economic status), recording of possible communication problems, whether the patient was at risk, number of contacts in the review year, urgency of the request for help, having seen more than one professional in the same practice, having seen one or more professionals outside the practice for the same health problem, the accuracy of the record keeping, and whether an incident had occurred. The primary care subgroups were free to add profession-specific questions.

For selected patient records in which an incident had occurred, the following items were added to the case registration form: a description of the incident (setting, incident, outcomes, judgement of the justification), and actions taken afterwards. The registration form was based on a form to be used in general practice care [28].

#### Incident-reporting study

We developed a structured form for reporting incidents that included the following items: type of incident, cause, actual harm to the patient, and probability of severe harm or death.

#### Survey

The questionnaire for practices addressed the following aspects: six questions about practice characteristics, 21 questions related to the presence of quality and safety management items (to be answered with 'yes' or 'no'), and 14 questions about the safety culture of the practice (on a five-point Likert scale).

The content of the questionnaire was derived from the *Visitation Instrument Accreditation *[29], the *Guidance for patient safety in general practice *[30], and the *Safety Attitudes Questionnaire *(SAQ, ambulatory version) [31]. The measures from the SAQ were translated systematically in a forward and backward translation procedure. If necessary, questions were adjusted to the type of healthcare practice.

### Data processing and data analysis

We analyzed the incidents found in the retrospective patient record study and the prospective incident-recording study by means of type of incident, causes, actual harm, and probability of severe harm or death. Types of incidents--not causes--are related to organization, environmental context (*e.g.*, materials and entrance), communication, prevention, triage, diagnostics, treatment, and/or intervention. We used the Eindhoven Classification Model [27] to classify the causes. We used the 'severity of outcome' domain of the International Taxonomy of Medical Errors in Primary Care [32] to define the severity level of the harm. We classed the probability of severe harm or death as 'very probable,' 'probable,' and 'not probable.' Table 3 gives an overview of the classifications.

**Table 3:** Overview of classifications

**Type of incident:**
Related to organization, communication, prevention, triage, diagnostics, and/or treatment.
**Cause(s) of the incident:**
Related to latent conditions (technical or organizational), active errors (human: knowledge-based behaviour, human: rule-based behaviour, human: skill-based behaviour), and other factors (patient related or other type) [27].
**Harm to the patient:**
Error, but no harm; error resulting in harm to the patient; error resulting in death; error, but harm indeterminate [32].
**Probability of severe harm or death:**
Very probable, probable, or not probable.

We used SPSS to enter the data in a database. In general, explorative analyses were involved. By this we mean that appropriate summary measures, such as mean and median values, were used. The accuracy of the figures was expressed in terms of 95% confidence intervals. Where necessary, we took into account the fact that the data were nested at the practice level. More details about analyses at the level of the sub-studies will be described in separate papers.

### Ethical approval/confidentiality (privacy)

According to the Dutch Central Committee on Research Involving Human Subjects regulations, only research in which the study participant has to be physically present during the study is subject to the Medical Research Involving Human Subjects Act [33]. Therefore, the committee stated in writing that ethical approval was not necessary. Each participating practice formally consented to participate.

Anonymity of practices, health professionals, and patients was and is of the utmost importance in this study. Several measures were taken to ensure the confidentiality of the collected information. The practices themselves selected the patient records and deleted any specific patient information, such as name, address, and date of birth. The reviewers signed a confidentiality agreement to maintain the secrecy of the information. The reviewers never reviewed in practices where they had ever been employed, and they did not and would never contact the individual patients or physicians. During the data collection, the records were never left unattended. Each record received a unique study number so that the patient's identity remained anonymous. Patient identifiers were kept in the practice and were destroyed on completion of the study.

If a reviewer had any concerns during the review process about unrecognized, potentially deliberate, harmful acts, illegal acts, or repetitive negligent behaviour, he would first of all discuss these concerns with the care provider. If doubt remained, the concerns could be further discussed with the internal ethics committee set up for this study.

### Timeframe

The complete study was planned to take place from January to December 2009. The part of the study described in this protocol was planned for May to December 2009.

## Discussion

There is no doubt that patient safety incidents occur in primary care. The aim of this study was to provide more detailed insight into the current patient safety issues in Dutch primary care in order to learn from current practice and to improve the quality of primary healthcare. It was difficult to estimate the frequency of the incidents. Much depended on the accuracy of the patient records and the lack of professionals' consensus regarding which types of adverse events were to be recognized as incidents. Gaining insight into the types, causes, and consequences of incidents was not too difficult. However, there was not enough information to do so in cases in which the healthcare professional did not realize that an incident had occurred. Hindsight bias comes into play in backward reviewing of patient records and incident-reporting forms [34,35]. In primary care, there are hardly any standardized registration or report systems for incidents. Substantial differences in record-keeping attitudes of professionals in primary care might have influenced the comparability of the results.

Another important factor is that the characteristics of the patient populations differ greatly across the practice types. For instance, in general dental care, most visits will be preventive. Physiotherapy care with a lot of elderly patients and many more contacts per patient, and midwifery care with many check-up visits contrast sharply with the immediate, symptomatically driven attendance at out-of-hours primary care centres. This has its implications for presenting results and probably for the type of follow-up research needed as well.

## Competing interests

The authors declare that they have no conflicts of interest.

## Authors' contributions

MH and MW developed the study protocol, while the other authors provided comments and helped to fit the protocol to specific factors in each professional environment to ensure that data collection and outcome measures would be sensible for unsafe situations. Moreover, all authors were involved in recruitment, data collection, data analysis, and interpretation of the data. MH drafted the manuscript with help from MW. All authors have read, commented on, and approved the final manuscript.

## Authors' information

Besides being experienced researchers, many of the authors are also actually working in primary healthcare. SG is a physician; SvD and RNvdS are physiotherapists; EdF, PG, and WV are GPs; LM is a midwife; and ThM is a GDP.
